# A Systematic Review and Meta-Analysis of Acupuncture Treatment for Oral Ulcer

**DOI:** 10.1155/2022/6082179

**Published:** 2022-11-08

**Authors:** Hang Yan, Tianxi Chen, Yuling Zuo, Yang Tu, Huangping Ai, Yuqi Lin, Yongcan Chen

**Affiliations:** ^1^Zhejiang Chinese Medical University, Hangzhou, Zhejiang, China; ^2^Hospital of Chengdu University of Traditional Chinese Medicine, Chengdu, Sichuan, China; ^3^Chengdu University of Traditional Chinese Medicine, Chengdu, Sichuan, China; ^4^Tongde Hospital of Zhejiang Province, Hangzhou, Zhejiang, China

## Abstract

**Background:**

Oral ulcer (OU) is a common oral mucosal disease manifested with obvious pain. In some studies, the efficacy of acupuncture in OU has been confirmed, but systematic reviews and meta-analyses for them are lacking. Our aim is to evaluate the efficacy of acupuncture in the treatment of OU.

**Methods:**

We searched the literature from eight databases from their inception to December 2021. We included randomized controlled trials of acupuncture for the treatment of oral ulcer. The meta-analysis was carried out using Review Manager 5.3 and Stata 16.0. The main outcomes were the effective rate and the recurrence rate, the secondary outcomes were the visual analogue score (VAS) and the ulcer healing time.

**Results:**

Totally, 18 studies were finally included in the meta-analysis, including 1,422 patients. In meta-analyses, we found that in comparison with Western medicine, acupuncture can improve effective rate (OR = 5.40, 95% CI: 3.40 to 8.58), reduce the ulcer recurrence rate (OR = 0.21, 95% CI: 0.13 to 0.33), and relieve the ulcer pain (MD = −2.26, 95% CI: −4.27to−0.24). In addition, compared with Western medicine, acupuncture plus Western medicine also can improve effective rate (OR = 2.95, 95% CI: 1.48 to 5.85). Compared with the Chinese medicine, the acupuncture plus Chinese medicine can improve the effective rate (OR = 8.26, 95% CI: 3.61 to 18.88) and relieve the ulcer pain (MD = −1.85, 95% CI: −2.51 to −1.19).

**Conclusion:**

Acupuncture may be more effective than Western medicine in terms of efficacy rate, and acupuncture combined with Western or Chinese medicine may have the potential to reduce the recurrence of ulcer and relieve the ulcer pain. However, due to limited evidence, higher quality and more rigorously designed clinical trials with larger sample sizes will be needed to further confirm our findings.

## 1. Introduction

Oral ulcer (OU) is the most common disease of oral mucosal all over the world [1, 2]. Among the gobal population, the incidence of OU is 5% to 20% [3, 4]. The clinical manifestations of OU are recurrent round or oval ulcers covered with yellow or grayish white pseudomembrane which are surrounded by a hyperemic red halo of about 1 mm. The central sunken of the ulcer has a soft base and obvious burning pain [5, 6]. Mild patients experience ulcer attack once a few months, while serious patients experience it all the time. Although the lesion does not bring serious damage to the body and can heal itself, the pain and discomfort caused by OU contribute unease to eating, drinking, teeth brushing, and even speaking, leading to a decline in the patient's quality of life and work efficiency [7, 8].

Presently, the etiology and pathogenesis of OU are not clear. Current studies believe pathogenic factors may include immune, gene, local trauma, mental stress, side effects of medicines, viral infections, and diet [9–12]. Hence, as a symptomatic treatment, OU therapy aims to relieve pain, promote ulcer healing, and extend the intermittent period of recurrence. The most commonly used medicines include glucocorticoids, growth factors, analgesics, anti-inflammatory drugs, mouth rinses containing active enzymes, and vitamins [13]. Unfortunately, the management of OU is still quite challenging with the undetermined efficacy of ulcer treatment. Brocklehurst found that no single therapy was effective enough for systemic intervention in OU [14]. At the same time, some treatment medicines also bring quite a few adverse reactions that are unbearable for patients, for example, long-term use of glucocorticoids may cause oral mucosal atrophy and immunity deficiency [15]. Therefore, it is necessary to develop new therapies with higher efficiency and lower side effects.

Acupuncture, a clinical subject that treats disease by stimulating acupoints on the body, is an important part of traditional Chinese medicine that runs thousands of years in East Asian countries. Meantime, as a complementary and alternative therapy, acupuncture has gained popularity in Western communities and the world at large. The 2007 National Health Interview Survey demonstrated that over 14 million Americans have used acupuncture as part of their health care, which was an increase from 8 million in 2002 [16]. The rise indicates that more individuals are accepting acupuncture treatment as part of their current health-care regimen. Acupuncture reduces pain by activating specific areas called acupoints on the patient's body. When these acupoints are fully activated, sensations of soreness, numbness, fullness, or heaviness called De qi or Te qi are felt by clinicians and patients [17, 18]. Recent studies have revealed that acupuncture can exert anti-inflammatory and analgesic effects by regulating peripheral (involving local acupoints and inflamed regions) and central neuroimmune interactions [19]. Due to aforementioned merits, acupuncture has been used successfully to treat migraine, knee and back pain, chemo-induced nausea, vomiting, and hot flash among other disorders [20]. In addition, acupuncture is often used to treat OU by relieving the pain of ulcer. Despite the numerous clinical research studies on acupuncture for OU, Wang et al. [21] showed that acupuncture can promote ulcer healing in patients with OU. Ren [22] believed that acupuncture can relieve the pain of OU. Liu [23] found that acupuncture can reduce the recurrence rate. Taking these inconsistent clinical outcome reports into account, a systematic evaluation is needed to summarize them to reach a consistent conclusion. Therefore, the aim of this systematic review and meta-analysis is to determine the effectiveness of acupuncture treatment for OU.

## 2. Methods

This system review had been registered on PROSPERO and the registration number was CRD42020144911.

### 2.1. Literature Search

Two researchers (Tianxi Chen and Yuqi Lin) conducted a comprehensive independent search on 8 electronic databases, including four English databases and four Chinese databases inceptions to December 2021. Four English databases including the Web of Science (WOS), PubMed, the Cochrane Library, and Embase; four Chinese databases including Chinese Biomedical Literature Database (CBM), Wanfang (WF), China Science and Technology Journal Database (VIP), and China National Knowledge Infrastructure (CNKI). The search terms were (“acupuncture”) AND (“mouth ulcer” OR “oral ulcer” OR “recurrent aphthous stomatitis”) AND (“randomized controlled trial”). The search strategy for PubMed is shown in Table 1.

### 2.2. Types of Study

The studies of acupuncture in the treatment of OU and the included studies were all randomized controlled trials (RCTs). There were no language or publication type restrictions. Quasi-RCTs and cluster RCTs were excluded.

### 2.3. Types of Participants

The inclusion criteria for participants were as follows: (I) participants who meet the diagnostic criteria of OU, regardless of their age, race, and gender. The exclusion criteria of participants were as follows: (I) participants who meet Behcet's disease, Reiter's syndrome, recurrent erythema multiforme, or any viral infection; (II) participants who are not suitable for acupuncture treatment, such as pregnant or lactating women and patients with other serious medical conditions.

### 2.4. Types of Interventions

Patients in the treatment group received conventional acupuncture, electroacupuncture, fire acupuncture, plum blossom acupuncture, press acupuncture, and other acupuncture therapies. There was no limit to the duration and frequency of treatment.

### 2.5. Types of Outcome Measures

The primary outcome measures assessed included the effective rate and the recurrence rate. Secondary outcome measures assessed included the visual analogue score (VAS) and the ulcer healing time.

### 2.6. The Risk of Bias Assessment

The risk of bias assessment of all studies in this review was independently assessed by two evaluators (Yang Tu and Huangping Ai) using RoB 2.0 tool published by the Cochrane Handbook. The following five items were assessed: randomization process, deviations from the intended interventions, missing outcome data, measurement of the outcome, and selection of the reported result. According to the RoB 2.0 guide, the signal problem of multiple module settings was judged. The signal problem answer was yes (Y), probably yes (PY), probably no (PN), no (no, N), or no information (NI). According to the signal answers given to different sections, the bias risk of each section was divided into low risk, some concerns, high risk, and the overall bias risk was given. If disagreement was seen in the assessments, this was resolved through discussion with a third researcher (Hang Yan).

### 2.7. Statistical Analysis

We conducted the meta-analysis using RevMan5.3 and Stata16.0 software. The odds ratio (OR) was used for dichotomous variables, mean difference (MD) and 95% confidence interval (CI) were used for continuous variables. We tested heterogeneity using the *I* square (*I*^*2*^) and *P* value (*P*). *P* < 0.1 or *I*^*2*^ > 50% was considered to indicate significant heterogeneity and was calculated using a random-effects model. Otherwise (*P* ≥ 0.1 or *I*^*2*^ ≤ 50%), the fixed-effect model was used, and the sources of heterogeneity were explored using subgroup analysis or sensitivity analysis.

### 2.8. GRADE Quality of Evidence Assessment

We used the GRADE profiler software to rank the quality of the evidence for the outcome indicators. GRADE identified five factors that may reduce the quality of evidence in interventional systematic reviews: risk of bias, inconsistency, imprecision, indirectness, and other considerations. The above five factors were evaluated by GRADE pro software, and the quality of evidence was divided into the following four levels: high, moderate, low, and very low, and the levels represented the strength of the evidence.

## 3. Results

### 3.1. Literature Search

**Figure 1:**
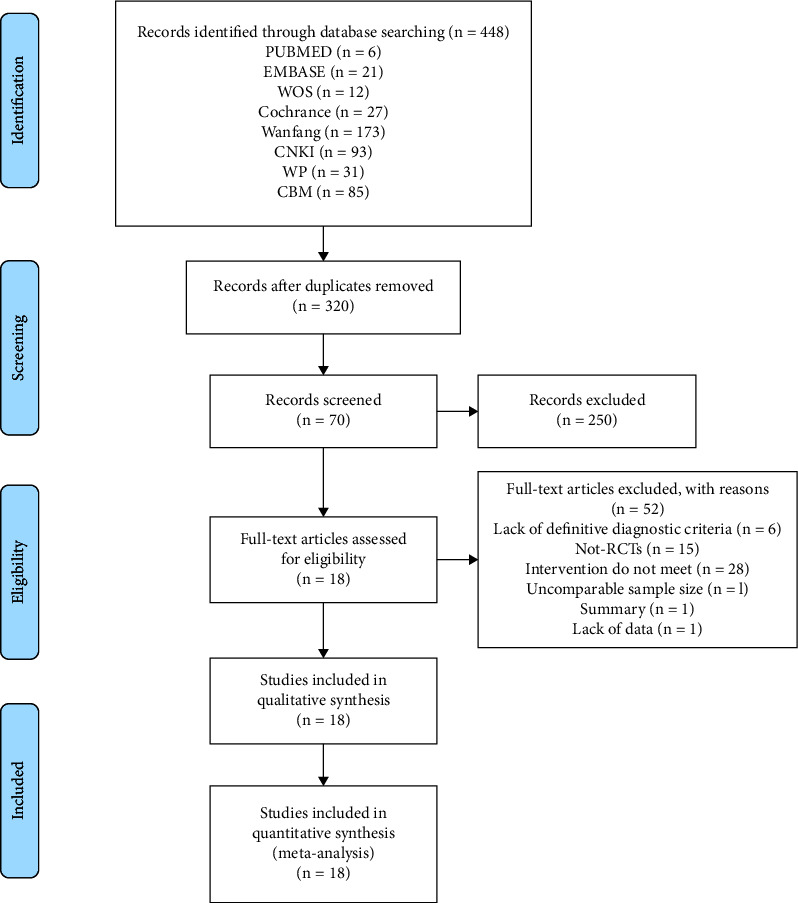
Flowchart of study selection process and screening results.

A total of 448 related articles were collected, including 6 from PubMed, 27 from Cochrane, 21 from Embase, 12 from WOS, 93 from CNKI, 173 from WF, 31 from WIP, and 85 from CBM. After excluding 128 duplicate literature, 320 RCTs remained. After initial screening, except for 250 articles, there were 70 articles left. After further full-text reading, 52 studies were excluded and 18 studies remained. The PRISMA flowchart of the literature search is shown in Figure 1.

### 3.2. Study Characteristics

We included 18 studies [24–41] with 1422 participants, 736 in the treatment group and 686 in the control group, all of which were published in Chinese between 2003 and 2021. Since there were many forms of acupuncture, such as electric acupuncture, fire acupuncture, screw acupuncture, moxibustion, and so on. All of them were considered as acupuncture in this study. The experimental intervention group included acupuncture, and the experimental control group included Chinese medicine and Western medicine. The included studies were divided into three groups based on the intervention in the experiment and the control groups: ① acupuncture versus Western medicine (*n* = 9) [24–32]; ② acupuncture plus Western medicine versus Western medicine (*n* = 4) [33–36]; ③ acupuncture plus Chinese medicine versus Chinese medicine (*n* = 5) [37–41]. Study characteristics of the included literature were summarized and listed in Table 2.

### 3.3. Risk of Bias in Included Studies

**Figure 2:**
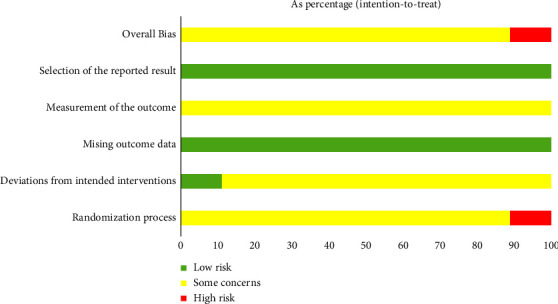
Graph of the risk of bias: percentage of all studies included.

**Figure 3:**
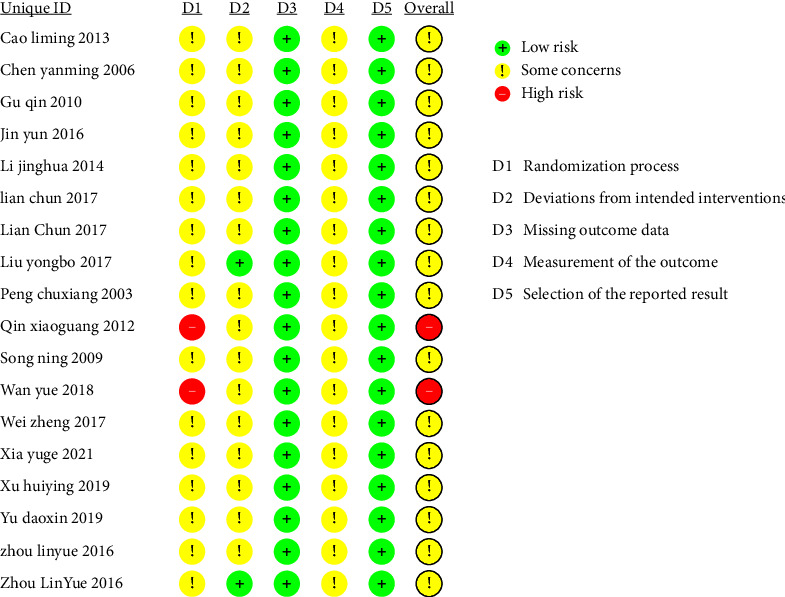
The ROB2 risk of bias.

Analysis of the included research trials according to ROB2 tool, 2 studies [24, 26] were assessed as high risk in domain 1 because they used random methods with a higher risk of bias. 2 studies [35, 37] were evaluated as low risk in domain 2 owing to mention blinding of participants. All included randomized controlled trials had low risk in domain 3 and domain 5. In terms of the overall risk of bias of the included studies, 2 studies [24, 26] were high risk and 16 [25, 27–41] studies were some concerns. The risk of the bias table is shown in Figures 2 and 3.

### 3.4. Analysis of the Effective Rate

**Figure 4:**
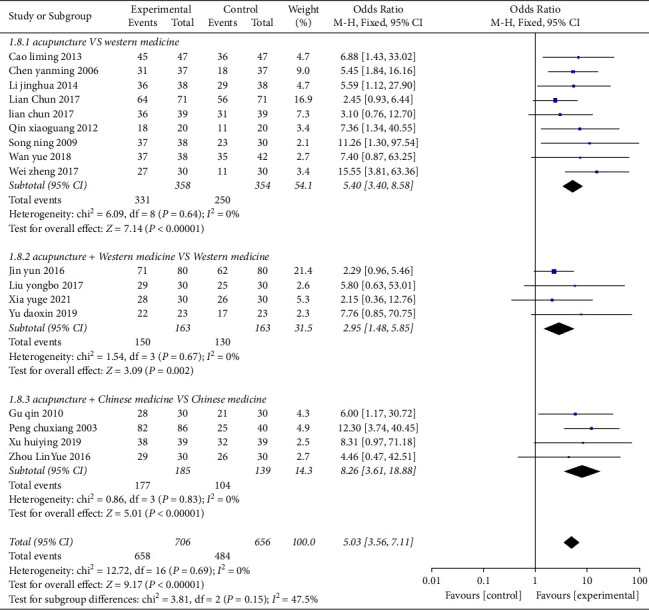
Forest plots of clinical efficacy rate in the three groups.

Seventeen studies [24–40] reported the effective rate involving a total of 1362 cases in Figure 4. Subgroup analysis showed that acupuncture can improve the effectiveness of OU regardless of its subtypes. After combining effect size, the OR value was 5.03 (95% CI: 3.56 to 7.11, *P* < 0.01, *I*^2^ = 0%). Nine studies [24–32] compared acupuncture with Western medicine, and the OR value was 5.40 (95% CI: 3.40 to 8.58, *P* < 0.01, *I*^2^ = 0%); it indicated that the effective rate of the acupuncture group was higher than that of the Western medicine group. Four studies [33–36] compared acupuncture plus Western medicine with Western medicine, and the OR value was 2.95 (95% CI: 1.48 to 5.85, *P* < 0.01, *I*^2^ = 0%); it indicated that the effective rate of acupuncture plus Western medicine group was higher than that of the simple Western medicine group. Four studies [37–40] compared acupuncture plus Chinese medicine with Chinese medicine, and the OR value was 8.26 (95% CI: 3.61 to 18.88, *P* < 0.01, *I*^2^ = 0%); it indicated that the effective rate of acupuncture plus Chinese medicine group was higher than that of the single Chinese medicine group.

### 3.5. Analysis of the Ulcer Recurrence Rate

**Figure 5:**
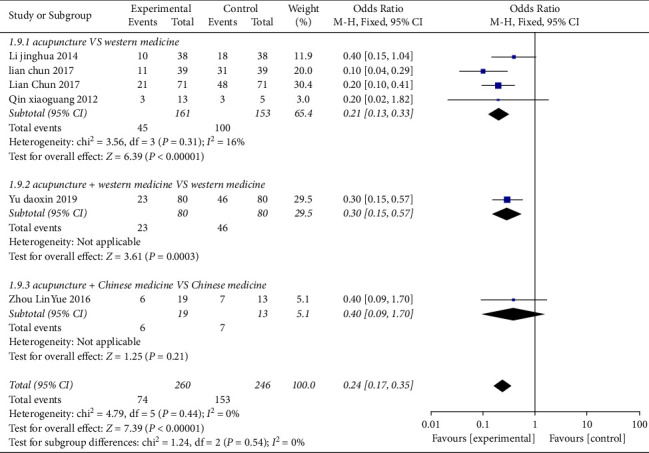
Forest plots of recurrence rate in the three groups.

Six studies [24, 28–30, 33, 37] reported the recurrence rate involving a total of 506 cases in Figure 5. Subgroup analysis showed that acupuncture can reduce the recurrence rate of OU regardless of its subtypes. After combining effect size, the OR value was 0.24 (95% CI: 0.17 to 0.35, *P* < 0.01, *I*^2^ = 0%). Four studies [24, 28–30] compared acupuncture with Western medicine, and the OR value was 0.21 (95% CI: 0.13 to 0.33, *P* < 0.01, *I*^2^ = 16%); it indicated that the recurrence rate of the acupuncture group was lower than that of the Western medicine group. One study [33] compared acupuncture plus Western medicine with Western medicine, and the OR value was 0.30 (95% CI: 0.15 to 0.57, *P* < 0.01); One study [37] compared acupuncture plus Chinese medicine with Chinese medicine, and the OR value was 0.40 (95% CI: 0.09 to 1.70, *P*=0.21).

### 3.6. Analysis of the Visual Analogue Score (VAS)

**Figure 6:**
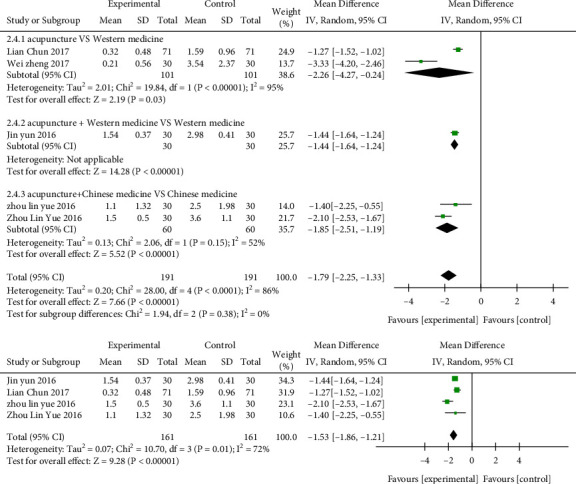
Forest plots of visual analogue score (VAS) in the three groups.

Five studies [25, 30, 34, 37, 41] reported the VAS indicator, involving a total of 382 cases in Figure 6. After combining effect size, the MD value was −1.79 scores (95% CI: −2.25 to −1.33, *P* < 0.01, *I*^2^ = 86%). Two studies [25, 30] showed that the visual analogue score in the acupuncture group was reduced by 2.26 scores compared with the Western medicine group (MD = −2.26, 95% CI: −4.27 to −0.24, *P*=0.03, *I*^2^ = 95%); one study [34] was acupuncture plus Western medicine compared with Western medicine, and the MD was −1.44 scores (95% CI: −1.64 to−1.24, *P* < 0.01); two studies [37, 41] showed that the visual analogue score in the acupuncture plus Chinese medicine group was reduced by 1.85 scores compared with the Chinese medicine group (MD = −1.85, 95% CI: −2.51 to−1.19, *P* < 0.01, *I*^2^ = 52%). After sensitivity analysis, it was found that one study [25] was the main source of heterogeneity. The heterogeneity was reduced after the removal of the study (*I*^2^ = 72%, *P* < 0.01).

### 3.7. Analysis of the Ulcer Healing Time

**Figure 7:**
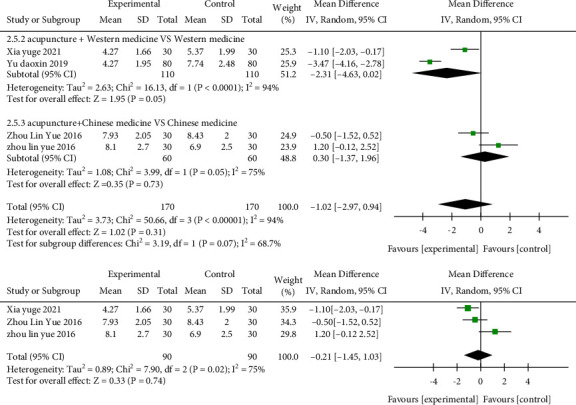
Forest plots of healing time in the three groups.

Four studies [33, 36, 37, 41] adopted the ulcer healing time as the outcome indicator in Figure 7, including a total of 340 cases. After combining effect size, the MD value was −1.02 days (95% CI: −2.97 to 0.94, *P*=0.31, I^2^ = 94%). Two studies [33, 36] showed that the ulcer healing time in the acupuncture plus Western medicine group was reduced by 2.31 days compared with the Western medicine group (MD = −2.31, 95% CI:−4.63 to 0.02, *P*=0.05, *I*^2^ = 94%); two studies [37, 41] was acupuncture plus Chinese medicine compared with Chinese medicine, and the MD was 0.30 days (95% CI: −1.37 to 1.96, *P*=0.73, *I*^2^ = 75%). After sensitivity analysis, one study [33] was the source of heterogeneity. The heterogeneity was reduced after the removal of the study (*I*^2^ = 75%, *P*=0.74).

### 3.8. Publication Bias Assessment

#### 3.8.1. Effective Rate Publication Bias

**Figure 8:**
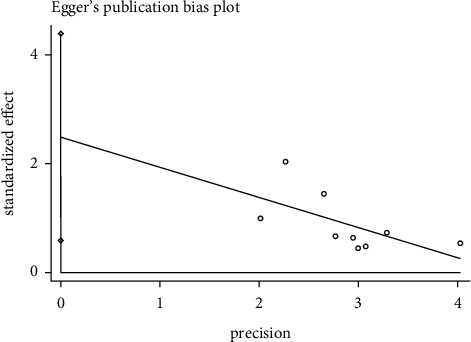
Egger's funnel plot for effective rate (acupuncture vs. western medicine).

**Figure 9:**
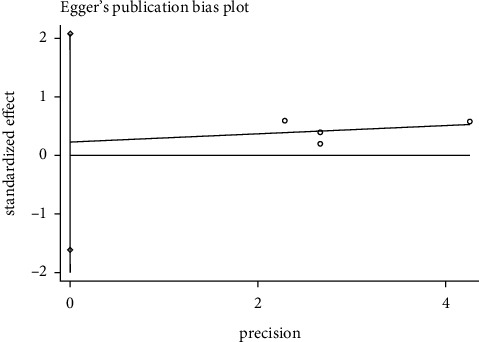
Egger's funnel plot for effective rate (acupuncture + western medicine vs. western medicine).

**Figure 10:**
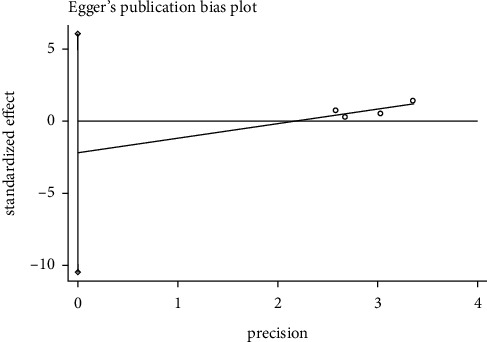
Egger's funnel plot for effective rate (acupuncture + Chinese medicine vs. Chinese medicine).

We used the Egger test to examine effective rate whether there was publication bias. In the acupuncture versus western medicine group, the result showed *P*=0.018 (*P* < 0.05), indicating that the 9 included articles had publication bias. In the acupuncture plus Western medicine versus Western medicine group, the outcomes showed *P*=0.643 (*P* > 0.05), meaning that there was no publication bias in the four included literature. In the acupuncture plus Chinese medicine versus Chinese medicine group, the results showed *P*=0.372 (*P* > 0.05), demonstrating that the four included literature did not have publications bias. It was shown in Figures 8–10.

#### 3.8.2. Recurrence Rate Publication Bias

**Figure 11:**
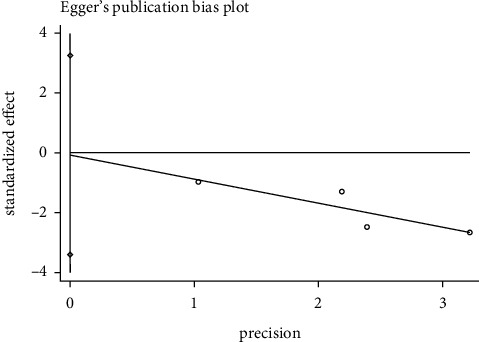
Egger's funnel plot for recurrence rate (acupuncture vs. western medicine).

Publication bias analysis was performed on the recurrence rate and the results showed that *P*=0.930 (*P* > 0.05), indicating that there was no publication bias in the acupuncture versus Western medicine group in Figure 11.

### 3.9. GRADE Quality of Evidence Assessment

We used GRADE pro 3.6 software to grade the quality of evidence for the 4 outcome measures: effective rate, recurrence rate, VAS, and healing time. The results showed that the effective rate and recurrence rate were low quality, the VAS and healing time were very low quality. The details are in Table 3.

## 4. Discussion

### 4.1. Summary of the Results

To the best of our knowledge, we expanded the scope of our search and found that there was still no significant evidence to support the effectiveness of acupuncture for OU in previous studies. Therefore, our study was the first systematic review and meta-analysis to evaluate the efficacy of acupuncture on the treatment of OU. This study adopted the Chinese Stomatological Association's trial criteria for evaluating the efficacy of oral ulcer [42]. The specific contents are as follows: the shortening of the average ulcer period and the pain index are markedly effective, the shortening of the average ulcer period or the pain index is effective, and the average ulcer period and the pain index which are not changed is invalid. We refer to the relevant content in the Guidelines for Clinical Research on New Chinese Medicines [43] that the recurrence rate of oral ulcer is three months after the end of treatment. The results of this study summarized the existing evidence on the efficacy of acupuncture in patients with oral ulcer till December 2021. We searched 8 Chinese and English databases, 18 RCTs with 1422 participants were reviewed in the meta-analysis. Compared with the Western medicine group, the acupuncture group can improve the effective rate (OR = 5.40, 95% CI: 3.40 to 8.58), reduce the ulcer recurrence rate (OR = 0.21, 95% CI: 0.13 to 0.33), and relieve the ulcer pain (MD = −2.26, 95% CI: −4.27 to −0.24). Compared with the Western medicine group, the acupuncture plus Western medicine group can increase the effective rate (OR = 2.95, 95% CI: 1.48 to 5.85). Compared with the Chinese medicine group, the acupuncture plus Chinese medicine group can improve the effective rate (OR = 8.26, 95% CI: 3.61 to 18.88), relieve the ulcer pain (MD = −1.85, 95% CI: −2.51 to −1.19). The above shows that the patients of OU could benefit from acupuncture therapy in terms of effective rate, ulcer recurrence rate, and visual analogue score. However, we performed the Egger test on the recurrence rate and effective rate. The results indicated that there was publication bias in the effective rate in the acupuncture versus Western medicine group. Meanwhile, the grade evidence results demonstrated that the effective rate and recurrence rate were low, the VAS score and healing time were very low. This suggested that we should be cautious in applying these results in clinical practice.

### 4.2. Limitations of the Results

There were some limitations of this research. First, the included 18 studies had methodological flaws and were assessed as being of low quality. We speculated for the following reasons. In the included literature, only two studies mentioned blinding of participants, and other studies did not clearly address randomization protocols, blinding methods, and allocation concealment, which may lead to selection, performance, and detection biases. Second, there was significant heterogeneity in the VAS and ulcer healing time as secondary outcome measures. We considered that both of these measures were subjective and easily influenced by the experience of clinicians and reviewers. Simultaneously, the number of clinical studies was less in two outcome measures. There were only five studies in the outcome measure of the VAS and four studies in the outcome measure of ulcer healing time, some results still need further confirmation. Third, we considered publication bias in the article. We used Egger's test to detect the effective rate and found that there was publication bias in the acupuncture versus Western medicine group. The specific reasons need to be further analyzed. Above all, the operation of acupuncture was subjective and some treatment standards were difficult to be unified. This study only focused on the stimulation method of acupuncture and did not analyze the differences in acupuncture point selection, manipulation depth, and intervention time. Next, the literature search strategy only searched 4 English databases and 4 Chinese databases, grey literature was not taken into consideration. Then, all the studies were published in China and there was potential publication bias in the included studies. Fourth, a subgroup analysis of ulcer classification would be more relevant in clinical practice, we did not perform a subgroup analysis of oral ulcer classification due to a small number of cases and incomplete data.

### 4.3. Suggestions for Future Studies

Based on the currently published evidence, this meta-analysis study shows that acupuncture is effective in the treatment of oral ulcer. However, some of the included studies have methodological flaws, which affect the authenticity, reproducibility, and comparability of research conclusions. It is not yet certain that acupuncture is completely superior to other treatments for oral ulcer. Therefore, we should formulate strict case inclusion and exclusion criteria and unified efficacy evaluation criteria, which have good feasibility. At the same time, the classification of ulcers has important implications for treatment options, and a subgroup analysis of ulcer classification is needed to clarify the efficacy of acupuncture on different ulcer subtypes in the future. Besides, specific acupuncture points, acupuncture stimulation methods, needle insertion depth, needle response, treatment course, qualifications of acupuncturists, assessors, and clinical practice years provide a rigorous, standard, and feasible treatment plan. Moreover, future efforts still need more high-quality, multicenter, large sample, randomized, double-blind, and placebo-controlled trials to improve the quality of the methodology and reporting.

## 5. Conclusion

In conclusion, the results of this systematic review suggest that acupuncture may be more effective than Western medicine in terms of efficacy rate, and acupuncture combined with Western or Chinese medicine may have potential to reduce the recurrence of ulcer and relieve the ulcer pain. However, due to limited evidence, higher quality and more rigorously designed clinical trials with larger sample sizes will be needed to further confirm our findings.

## Figures and Tables

**Table 1:** The detailed search strategy in PUBMED as an example.

Numbers	Search terms
^#^1	Recurrent aphthous stomatitis
^#^2	Canker sore
^#^3	Recurrent aphthous ulcer
^#^4	Recurrent oral ulcer
^#^5	Oral ulcer
^#^6	Mouth ulcer
^#^7	Aphthous stomatitides
^#^8	Aphthous stomatitis
^#^9	Aphthous ulcer
^#^10	Aphthae
^#^11	Recurrent stomatocace
^#^12	OR/^#^1-^#^11
^#^13	Meridian*∗*
^#^14	Acupressure
^#^15	Warm needling
^#^16	Moxa needle
^#^17	Auricular acupuncture
^#^18	Auricular needle
^#^19	Acupuncture
^#^20	Acupuncture therapy
^#^21	Electroacupuncture
^#^22	Electroacupuncture therapy
^#^23	Manual acupuncture
^#^24	Dry needle
^#^25	Moxibustion
^#^26	Acupoint
^#^27	Ear acupuncture
^#^28	Abdom *∗* acupuncture
^#^29	Embed *∗* thread therapy
^#^30	Catgut embedding
^#^31	OR/^#^13-^#^30
^#^32	Randomized controlled trial
^#^33	Controlled clinical trial
^#^34	Randomized
^#^35	Randomized
^#^36	Placebo
^#^37	Randomly
^#^38	Trial
^#^39	Groups
^#^40	OR/^#^32-^#^39
^#^41	^#^12 AND ^#^31 AND ^#^40

**Table 2:** Characteristics and details of interventions of included studies.

Studies	Year	Sample sizes (E/C)	Age		Gender (male/female)		Duration		Intervention		Length of treatment	Outcomes
			Experimental	Control	Experimental	Control	Experimental	Control	Experimental	Control		
Qin Xiaoguang	2012	40 (20/20)	7.5 ± 2.6	6.9 ± 2.3	11/9	13/7	2.6 ± 1.1D	2.8 ± 1.0D	A	WM	7D	ER + RR
Wei Zheng	2017	60 (30/30)	59.33 ± 6.88	62.66 ± 7.67	13/17	15/15	15.57 ± 7.09D	15.97 ± 6.70D	A	WM	20D	ER + VAS
Wan Yue	2018	80 (40/40)	51.38 ± 8.58	52.02 ± 9.26	20/18	21/21	10.51 ± 3.04Y	11.04 ± 4.39Y	A	WM	28D	ER
Chen Yanming	2006	74 (37/37)	35.22 ± 3.28	35.30 ± 2.86	21/16	14/23	8.8 ± 3.8Y	8.6 ± 3.2Y	A	WM	14D	ER
Li Jinghua	2014	76 (38/38)	Not described	Not described	Not described	Not described	Not described	Not described	A	WM	20D	ER + RR
Lian Chun	2017	78 (39/39)	42.27 ± 7.79	43.38 ± 8.03	23/16	21/18	18.24 ± 5.32 M	17.89 ± 6.13 M	A	WM	30D	ER + RR
Lian Chun	2017	142 (71/71)	38.97 ± 8.47	40.02 ± 7.68	47/24	45/26	11.04 ± 3.51 M	11.39 ± 4.03 M	A	WM	30D	ER + RR + VAS
Cao Liming	2013	94 (47/47)	15–67	15–67	Not described	Not described	Not described	Not described	A	WM	7D	ER
Song ning	2009	68 (38/30)	16–55	18–60	17/21	12/18	6M-6y	3M-8y	A	WM	14D	ER
Yu Daoxin	2019	160 (80/80)	38.60 ± 9.46	37.85 ± 8.92	37/43	33/47	1.48 ± 2.10Y	1.63 ± 2.44Y	A + WM	WM	30D	ER + RR + UHT
Jin Yun	2016	60 (30/30)	63 ± 9	64 ± 9	10/20	12/18	2.31 ± 0.55Y	2.27 ± 0.49Y	A + WM	WM	5D	ER + VAS
Liu Yongbo	2017	46 (23/23)	18–61	18–61	Not described	Not described	6M-21y	6M-21y	A + WM	WM	14D	ER
Xia Yuge	2021	60 (30/30)	39.27 ± 12.70	38.90 ± 13.74	11/19	14/16	13.37 ± 6.64Y	15.87 ± 8.14Y	A + WM	WM	6D	ER + UHT
Zhou LinYue	2016	60 (30/30)	42.70 ± 14.65	43.50 ± 14.31	17/13	20/10	42.70 ± 14.65Y	43.50 ± 14.31Y	A + CM	CM	7D	ER + RR + VAS + UHT
Zhou Lin Yue	2016	60 (30/30)	Not described	Not described	Not described	Not described	Not described	Not described	A + CM	CM	7D	VAS + UHT
Xu Huiying	2019	78 (39/39)	41.58 ± 3.27	8.94 ± 1.36	23/16	22/17	41.58 ± 3.27Y	8.94 ± 1.36Y	A + CM	CM	6D	ER
Gu Qin	2010	60 (30/30)	13–70	12–68	13/17	20/10	Not described	Not described	A + CM	CM	14D	ER
Peng Chuxiang	2003	126 (86/40)	40.1 ± 5.6	38.5 ± 6.2	35/51	17/23	8.6 ± 3.2Y	8.8 ± 3.8Y	A + CM	CM	60D	ER

A: acupuncture, WM: western medicine, CM: Chinese medicine, A + WM: acupuncture + western medicine, A + CM: acupuncture + Chinese medicine, D: day, M: month, Y: year, ER: effective rate, RR:r ecurrence rate, VAS: visual analogue score, and UHT: ulcer healing time.

**Table 3:** GRADE evidence quality of outcomes included in the literature.

Certainty assessment								Effects		Quality
Outcomes	No. of studies	Design	Risk of bias	Inconsistency	Indirectness	Imprecision	Other considerations	Relative (95% CI)	Absolute (95% CI)	
The effective rate	17	RCT	Serious^a^	No serious inconsistency	No serious indirectness	No serious imprecision	Publication bias^d^	OR 5.03 (3.56 to 7.11)	196 per 1,000 (from 171 to 215)	⊕⊕○○ low
The ulcer recurrence rate	6	RCT	Serious^a^	No serious inconsistency	No serious indirectness	Serious^b^	None	OR 0.24 (0.17 to 0.35)	339 fewer per 1,000 (from 403 fewer to 257 fewer)	⊕⊕○○ low
Visual analogue score	5	RCT	Serious^a^	Serious^c^	No serious indirectness	Serious^b^	None	—	MD 1.79 SD lower (2.25 lower to 1.33 lower)	⊕○○○ very low
The ulcer healing time	4	RCT	Serious^a^	Serious^c^	No serious indirectness	Serious^b^	None	—	MD 1.02 SD lower (2.97 lower to 0.94 higher)	⊕○○○ very low

Explanations. (a) Some study randomization methods, allocation concealment, and blinding are not described. (b) Fewer included articles and observers. (c) Heterogeneity is significantly higher. (d) Publication bias.

## Data Availability

The data of this study are obtained from open databases, and the data used in the study have been submitted in the manuscript.

## Supplementary Materials

Supplementary Materials The search strategy of PUBMED database as an example.
