# Supplementary material for: A Systematic Review and Meta-Analysis of Acupuncture Treatment for Oral Ulcer
Source: Evid Based Complement Alternat Med. 2022 Nov 8;2022:6082179. doi: 10.1155/2022/6082179 (PMC9666018; doi:10.1155/2022/6082179)
Supplement: Supplementary Materials — The search strategy of PUBMED database as an example. [file 6082179.f1.docx]

Take the search strategy of PUBMED database for example

| Number | Search terms |
| --- | --- |
| #1 | recurrent aphthous stomatitis |
| #2 | canker sore |
| #3 | recurrent aphthous ulcer |
| #4 | recurrent oral ulcer |
| #5 | oral ulcer |
| #6 | Mouth Ulcer |
| #7 | Aphthous Stomatitides |
| #8 | Aphthous Stomatitis |
| #9 | Aphthous Ulcer |
| #10 | Aphthae |
| #11 | recurrent stomatocace |
| #12 | OR/#1-#11 |
| #13 | meridian* |
| #14 | acupressure |
| #15 | warm needling |
| #16 | moxa needle |
| #17 | auricular acupuncture |
| #18 | auricular needle |
| #19 | acupuncture |
| #20 | acupuncture therapy |
| #21 | electroacupuncture |
| #22 | electroacupuncture therapy |
| #23 | manual acupuncture |
| #24 | dry needle |
| #25 | moxibustion |
| #26 | acupoint |
| #27 | ear acupuncture |
| #28 | abdom*acupuncture |
| #29 | embed*thread therapy |
| #30 | catgut embedding |
| #31 | OR/#13- #30 |
| #32  #33  #34  #35  #36  #37  #38  #39  #40  #41 | randomized controlled trial  controlled clinical trial  randomized  randomised  placebo  randomly  trial  groups  OR/#32-#39  #12 AND #31 AND #40 |
